# Medical Opinion and Movement

**Published:** 1908-04-04

**Authors:** 


### Medical Opinion and Movement.

EXOPHTHALMIC goître is one of those diseases in the treatment of which nearly everything has been tried. Many have appeared to be more or less efficacious in a few cases, but in none has a specific cure been discovered. It does not seem likely that quinine has not also received a trial for this condition. It is rather surprising, therefore, to find that MM. Lancereaux and Paulesco have recently advocated quinine as a specific for Grave's disease in a paper read before the Academy of Medicine. The idea occurred to them to use this drug on the ground that it is a typical vaso-constrictor, and they regard as the chief pathological feature of exophthalmic goître a vaso-dilatation of the vessels of the head and neck. They have used the sulphate in doses from 1 gramme to $1\frac{1}{2}$ grammes in cachets of 50 centigrammes every evening with food. The treatment was continued for several months with occasional intervals, and the results have been highly satisfactory. They have now treated more than twenty cases. The phenomena of vaso-dilatation have disappeared, and later the other chief symptoms—the tachycardia, the exophthalmos and the goître itself. In other words, they claim to have effected a complete recovery. It will be interesting to hear if the experiences of others confirm these surprising results.

DELAYED chloroform poisoning in children has attracted considerable attention on account of several cases of this condition that have recently been published. The pathology of the condition is still very obscure, and the treatment correspondingly unsatisfactory. The presence of acetone in the breath and in the urine, accompanied by more or less coma suggests an acid intoxication similar to the last stages of diabetes. Post-mortem examination invariably shows an accumulation of fat in the liver cells, and to a less degree in the cells of the muscles and kidneys. The acid-intoxication theory naturally suggested the administration of alkalies without, however, any very satisfactory results. There is no doubt that in these cases the cells of the body are directly poisoned by the chloroform, and experiments on animals have shown that the action of chloroform on the cells is similar to that of phosphorus, arsenic, and other general protoplasmic poisons. Why chloroform in ordinary doses should have this effect in certain individual cases remains at present the chief mystery in the condition. Rosenfeld explains the intoxication as in the nature of a starvation of the tissues owing to a change in their metabolic powers. They can utilise carbohydrates, but can only oxidise proteids and fats imperfectly. The store of carbohydrates is quickly used up, and their hungry condition causes a breaking down of tissue proteid and a transference of fat to them. Since they cannot utilise these foodstuffs properly they remain in a condition of starvation, which may rapidly lead to death. Dr. Beddard, in adopting this hypothesis, suggests that the appropriate treatment might be the administration of carbohydrates in the form of enemata or infusions of 10 or 20 per cent. solutions of dextrose. Feeding with carbohydrates could not prevent the poison from damaging the cells, but it might supply them with a necessary source of energy, and so prevent death from acute inanition.

GASTROSCOPY, by means of an instrument introduced through the mouth and œsophagus, has not proved of any great utility owing to the incomplete view obtained through such a long tube. Professor Thorkild Rovsing has, however, devised a method of performing gastroscopy and gastrodiaphanoscopy in cases where, even after laparotomy, the diagnosis is still doubtful. The instrument, similar to an ordinary cystoscope, but larger, is introduced through a small aperture made in the anterior wall of the stomach. The stomach is inflated by pumping air through a channel in the instrument, and the viscus is then trans-illuminated from within by means of the little lamp of the instrument. In normal cases the organ appears as a yellow globe, but in cases of gastritis it appears red. Any infiltration is shown as a dark spot. The examination is completed by gastroscopy, the optical apparatus of the instrument allowing a clear view of the interior of the stomach. It is claimed that the use of the instrument will facilitate the detection of the bleeding spot in cases of obscure hæmatemesis.

DR. F. J. WALDO, Coroner for the City of London and Borough of Southwark, has recently inquired into the question of the mortality due to general anæsthetics, and shows conclusively that the present available data are so imperfect as to be useless for the purpose of formal investigation. A consideration of the annual reports of the Registrar-General for England and Wales and the source from which these are obtained demonstrates their imperfections both in regard to numbers and details. Thus during the years 1902-6 there was a total of 786 deaths reported, and of these in 204 cases—about 25 per cent.—the precise drug used is not reported. A more serious contention, however, is that the returns fail to show the full number of deaths actually due to this cause. The sources from which the information is obtained are the copies of verdicts at inquests and the certificates of medical practitioners. Dr. Waldo points out that, in spite of the recommendation of the Coroners' Society, it is not a universal custom of coroners to hold inquests upon deaths reported to them to have occurred under anæsthesia. Furthermore, owing to the disproportion of total cases in hospital and private practice, he is led to the conclusion that fatal cases occurring in private practice are often otherwise certified. In order, therefore, to ensure more proper supervision and obtain satisfactory conclusions as to the precise facts of all deaths under anæsthetics, Dr. Waldo makes certain recommendations. Chief among these are that public institutions should appoint skilled anæsthetists to administer or supervise the administration of anæsthetics, and that full details of all administrations should be recorded; that all deaths occurring at any stage of general anæsthesia should be notified to the coroner, and that coroners be required to hold a public inquiry on all such deaths and make a detailed report to the Registrar-General, together with the verdict.

In a recent issue of The Hospital we drew attention to the desirability of studying the diseases of historical characters. The Paris Academy of Medicine has on more than one occasion during the present session had before it a communication or paper dealing with the clinical biography of one or other celebrated French writer. A month ago an interesting discussion was evoked by the reading of a critical and psychologically-analytical elucidation of Chateaubriand. Now, again, M. Armengaud has dealt with Montaigne, and at one of its February meetings the Academy considered "Montaigne as hypochondriac." It is satisfactory to find that the great French essayist was exonerated from all hypochondriacal tendency. Montaigne, indeed, is one of those who has suffered at the hands of the would-be-medical biographer, and a great deal of nonsense has been written about his state of body and mind. It was high time for the faculty to whitewash him, and M. Armengaud has done the whitewashing with skill and not a little literary ability, so that the reading of the paper was followed with lively interest.

Zweifel, in the "Munchener Med. Wochenschrift," discusses the treatment of placenta prævia in private practice. He considers that every severe hæmorrhage during the second half of pregnancy is to be regarded as highly suggestive of placenta prævia and to be treated accordingly. Plugging and artificial rupture of the membranes are the best methods for inducing labour, but the latter is rarely possible. Combined version is one of the best and most certain ways of treating the hæmorrhage. It must be performed with the strictest aseptic precautions. In central cases an attempt should first be made to displace the flap of the placenta with two fingers, and if that fails to bore through the placental tissue itself; the author condemns Cæsarean section in cases of placenta prævia.

There is a certain amount of daring in attacking a problem which has engaged the attention of the foremost surgeons of the day for more than a century, but the presumption of "a mere anatomist" may be forgiven if it produces so excellent a result as Mr. A. R. Thompson's Hunterian lectures on "The Anatomy of the Long Bones in connection with certain Fractures." In the first lecture Mr. Thompson, who is one of the demonstrators of anatomy and late surgical registrar at Guy's Hospital, chiefly dealt with fractures about the neck of the femur. Intra- and extra-capsular fractures have been subjects of profound interest in the past, and no one can honestly say that we have still little to learn about their ætiology, incidence, and treatment. It has been pointed out, chiefly by French writers, that the pressure and tension lamellæ in the shaft and upper end of the femur are considerable factors in determining the direction and extent of fractures in this bone, but no one as yet has so forcibly put the case for the calcar femorale as Mr. Thompson did in his initial lecture. According to him the calcar, that strong mass of compact pressure lamellæ which is found in the upper end of the thigh bone, acts, directly almost, as a chisel in producing fractures of the neck, splitting the bone to produce the so-called extra-capsular variety. Illustrating his remarks with diagrams and really helpful models, he went on to show how the arrangement of pressure and tension lamellæ modified the fracture, and how the deformities found at the hip-joint are produced. Incidentally he suggested a new nomenclature. Everyone knows the objections to the old terms, which are misleading and mistaken from an anatomical point of view. No fracture of the neck is really intra-capsular, strictly speaking, and the extra-capsular variety again is never entirely without the limits of the capsule. Bigelow and Redus have suggested a modified terminology, but their coinages have not met with much favour. Mr. Thompson now proposes the words paracephalic and paratrochanteric for the old terms intra-capsular and extra-capsular. The suggestion is worth consideration, but we do not think that the profession will give up the old, though misleading favourites, for the new terms proposed by "a mere anatomist."

Garnier, in the course of a clinical lecture at the Hopital Broussais, discussed the prognosis in lesions of the liver due to the action of alcohol. Leudet in 1860 described what he called "benign alcoholic jaundice" in contrast to the alcoholic icterus of other authors. In both varieties, which are, after all, merely symptoms of acute or sub-acute alcoholic intoxications, the liver cells are involved, but the prognosis in the former lesion is much better than in the latter, in which the hepatic inflammation is usually grave, leading often to a fatal issue. Gilbert and Lereboullet in 1902 described a latent parenchymatous fatty degeneration of the liver, which is clinically evident by few signs pointing specially to the liver. The patients usually die of intercurrent diseases, pneumonia being especially fatal in such patients. The prognosis in "steatose lasense" is very bad, but is not necessarily fatal, for the patient can always be cured if the diagnosis is made early, and if all alcohol is prohibited forthwith. Incurable, on the other hand, is the ordinary form of fatty alcoholic cirrhosis, of which, in accordance with all French authorities, M. Garnier recognises two varieties—the hypertrophic form of Hutinel and the atrophic form which corresponds to the atrophic cirrhosis described by Hands and Gilbert as a "chronic tuberculous atrophic hepatitis." In both cases the lesion is parenchymatous and the prognosis is grave. In the interstitial cirrhotic conditions the prognosis varies. The author discusses the three main types of the interstitial lesion—diffuse alcoholic cirrhosis, hypertrophic alcoholic cirrhosis (not to be confounded with the parenchymatous hypertrophic form), and the atrophic form, or Lænnec's disease. The first was described by Gilbert and Garnier in 1897, and is a rapidly fatal disease, in which the prognosis is always unfavourable. Lænnec's cirrhosis is an equally serious condition, though it is much less rapid in its course, the patients usually living two or more years after the onset of the disease. The prognosis in the hypertrophic form is the most favourable. It is slightly less favourable in the pigmentary variety of this type, the so-called pigmentary hypertrophic alcoholic cirrhosis of Letulle, and much more favourable on the contrary in the "anascitic cirrhosis" of Lereboullet, which is quite a benign form. Still another form is the so-called "diabetic," described by Gilbert and Lereboullet, in which glycosuria is a prominent symptom, and in which the prognosis is always grave. That alcohol is the cause of all these varieties of parenchymatous and interstitial lesions, some of which can hardly be accepted as clinical entities, is still to be proved. M. Garnier's lecture does not throw much light on the ætiology, and leaves the question of treatment entirely alone. As a summary of liver lesions presumably due to the effects of alcohol, it is, however, excellent.

---

Sternberg has found in the ligamentum nuchæ an excellent adjuvant in the treatment of corpulence and obesity. Patients who are ordered a strict diet usually complain that their appetite is never satisfied, and the same complaint is made by those who, being used to a mixed diet, are placed on a physiologically equivalent vegetable diet. A fish diet is always less "filling" than a flesh diet, and the latter again less so than a diet of eggs, weight for weight. As a general rule it may be stated that compact food is more satisfying than liquid or semi-solid diet. Sternberg recommends the ligamentum nuchæ of the ox, which has a very low dietetic value, as a satisfactory adjuvant to such "non-filling" diets. It is carefully cleaned from all fat or other tissue, and is then boiled for several hours in normal saline solution until it is quite soft. It can then be minced very finely in a mincing machine, and can be dried into a fine grey powder. This powder can be made into tablets or into small loaves, even without the addition of flour. Such ligamentum nuchæ loaves are appetising and filling, though they have but little value as food. In diabetic diet they are specially useful, and in diets for obesity they are always relished. The ligamentum nuchæ is usually thrown away, and can be procured from most butchers for the asking.

---

Hildebrand, in a recent article on the surgery of the œsophagus, lays stress on the fact that although in recent years the diagnosis of œsophageal lesions has been greatly improved and modified by the use of the œsophagoscope, the treatment of such lesions remains in a very unsatisfactory state. It is true that carcinomata of the upper end are not infrequently completely extirpated. Hildebrand himself has operated on six cases, one of his patients who was operated upon three years ago being still alive and without recurrence. The operation is, however, a very serious one, the mortality being unusually high. Much more serious and much more open to improvement are the operative methods for lesions in the middle and at the lower end of the œsophagus. Pure œsophagotomy, for the removal of foreign bodies low down, is a difficult operation; gastrotomy is usually preferred, and the impacted body pushed lower down and removed through the stomach. In order to facilitate such extraction Killian and Mikulicz have attempted to perforate foreign bodies by a fine œsophageal sound, which is connected with an electric battery, insulated except at the extremity, and thus made to act as an efficient cautery. Discussing the various proposed methods for extirpation of growths whose site is the lower end of the œsophagus, Hildebrand is of opinion that they offer very little hope of successful accomplishment. Such growths are diagnosed relatively late, when the patient's chances of being radically cured are infinitesimal, and when the dangers and difficulties of the operation are enormously increased owing to the encroachment of the growth upon surrounding parts. Sauerbruch's cabinet, which has done such good service in operations on the lungs and pleuræ, does not make the operation on the thoracic part of the œsophagus any easier, and up to the present no successful case of thoracic œsophagectomy has been published. The dangers of periœsophageal abscesses, of empyema, and of lesions to nerves and vessels are very great. There is no doubt that it is possible to suture the stump of the œsophagus successfully, but it is very doubtful if the sutures hold. Hitherto, owing to the fact that no patient has survived the operation for any length of time, it is impossible to discuss the question of the efficiency of proposed methods of suture. The author thinks the operation of thoracic œsophagectomy is therefore unjustifiable, and should be given up in favour of the older methods of palliative gastrostomy.

---

Leuzmann of Duisburg has published the results in a series of cases of syphilis treated by a new method. This method consists essentially in bringing the patient rapidly under the influence of quinine. Intravenous injections of 0.5 to 0.8 gramme of quinine hydrochloride are given, the salt being dissolved in normal saline. Four c.c. of this solution, which has been previously warmed, are injected into the veins of the arm or leg. Where very stout persons in whom such intravenous injections are exceedingly difficult to carry out, intramuscular or even subcutaneous injections may be given. It is advisable to commence the treatment with a relatively small dose—usually 0.3 gramme—and to give one injection per day. After four days' treatment injections are only given every second and later every fourth day. Fourteen to twenty injections are usually sufficient. The results obtained have been exceedingly encouraging. More especially striking has been the rapid improvement leading to complete cure which the author was able to obtain in several serious cases of so-called malignant syphilis. Good results were also obtained in cases of secondary and tertiary syphilis and in primary chancres. The method is still on its trial, but the results which have been published are so striking that the treatment is worth careful consideration by the profession generally.

